# Maritime transport and regional climate change impacts in large EU islands and archipelagos

**DOI:** 10.1007/s41207-023-00370-6

**Published:** 2023-05-26

**Authors:** George Zittis, Bodo Ahrens, Anika Obermann-Hellhund, Elias Giannakis, Danny Risto, Miguel Agulles Gamez, Gabriel Jorda, Mónica Quesada Peña, Veronica Lora Rodríguez, Jose Luis Guersi Sauret, Piero Lionello, Elodie Briche, Julie Collignan, Matthias Grätz, Damian Arikas, Constantinos Stylianou, Haris Neophytou, Despina Serghides

**Affiliations:** 1 Climate and Atmosphere Research Center (CARE-C), The Cyprus Institute, Nicosia, Cyprus; 2 Institute for Atmospheric and Environmental Sciences (IAU), Goethe University Frankfurt, Altenhoeferallee 1, 60438 Frankfurt, Germany; 3 Energy, Environment and Water Research Center (EEWRC), The Cyprus Institute, Nicosia, Cyprus; 4 Mediterranean Institute for Advanced Studies (IMEDEA, UIB-CSIC), Esporles, Spain; 5 Centro Tecnológico de Ciencias Marinas (CETECIMA), Telde, Spain; 6 Dipartimento di Scienze e Tecnologie Biologiche ed Ambientali, University of Salento, Lecce, Italy; 7 Euro-Mediterranean Center on Climate Change (CMCC), Lecce, Italy; 8 Agence de la Transition Écologique (ADEME), Angers, France; 9 Laboratoire de Météorologie Dynamique/IPSL-Ecole Polytechnique, Paris, France; 10 Baltic Environmental Forum Deutschland (BEF), Hamburg, Germany; 11 Interfusion Services (IF), Limassol, Cyprus; 12 Department of Agricultural Economics and Rural Development, Agricultural University of Athens, Athens, Greece

**Keywords:** Climate change, Impacts, Maritime transport, Blue economy, Mediterranean, Islands

## Abstract

Maritime transport is a vital sector for global trade and the world economy. Particularly for islands, there is also an important social dimension of this sector, since island communities strongly rely on it for a connection with the mainland and the transportation of goods and passengers. Furthermore, islands are exceptionally vulnerable to climate change, as the rising sea level and extreme events are expected to induce severe impacts. Such hazards are anticipated to also affect the operations of the maritime transport sector by affecting either the port infrastructure or ships en route. The present study is an effort to better comprehend and assess the future risk of maritime transport disruption in six European islands and archipelagos, and it aims at supporting regional to local policy and decision-making. We employ state-of-the-art regional climate datasets and the widely used impact chain approach to identify the different components that might drive such risks. Larger islands (e.g., Corsica, Cyprus and Crete) are found to be more resilient to the impacts of climate change on maritime operations. Our findings also highlight the importance of adopting a low-emission pathway, since this will keep the risk of maritime transport disruption similar to present levels or even slightly decreased for some islands because of an enhanced adaptation capacity and advantageous demographic changes.

**Supplementary Information:**

The online version contains supplementary material available at 10.1007/s41207-023-00370-6.

## Introduction

Maritime transport is defined as the carriage of goods and passengers by sea-going vessels on voyages undertaken wholly or partly at sea. It is often considered the backbone of the world economy, with 80% of the global trade volume passing through ports (Asariotis and Benamara 2012). At the same time, the sector itself contributes to global warming through its carbon emissions, which are found to be nearly 3% of the global CO_2_-equivalent emissions (IMO 2015). Nevertheless, compared to land and air transport, it is by far the most cost-effective means of distributing goods globally. Ship emissions constitute a significant and, so far, poorly regulated source of air pollutants, including sulfur dioxide, NOx, and primary particles, which can also lead to the formation of fine secondary particles (PM2.5), acidification and eutrophication (Jonson et al. 2020). A changing climate is therefore expected to challenge maritime transport to adapt to future risks and, at the same time, substantially lower its emissions to meet the global decarbonization targets (e.g., Gütschow et al. 2021).

Maritime transport is one of the key European Union (EU) Blue Economy sectors, since Europe is amongst the leading maritime centers in the world, with more than 300 major seaports along its coastline and control of around one-third of the world’s merchant fleet.^1^ For EU countries, ports are vital gateways linking European transport corridors to the rest of the world. As 75% of European external trade transits through EU ports, the shipping sector plays a significant role in connecting the European market with its trade partners. For some Mediterranean countries, including Cyprus, Greece and Malta, there is a significant direct contribution (3–10%) of the maritime transport sector to the total gross value added (GVA) (Eurostat 2020). Giannakis et al. (2019) also highlight the strong backward linkages of the water transport sector in the EU economy. For example, in 2015, for every 1 million euro increase in the final demand for the products and services of this industry, the total economic output increased by 2 million euros (Giannakis et al. 2019). However, for every 1 million euro increase in the final demand for the products and services of this sector, 23.7 metric tons of NOx, 8.5 tons of SOx, 2 tons of PM10 (particles with diameters of 10 microns or less) and 1.8 tons of PM2.5 (particles with diameters of 2.5 microns or less) are emitted, while the sector already creates the largest direct and indirect emissions across the EU. According to Eurostat (2020), the sector employs nearly 250,000 persons (or 0.1% of the EU-27 workforce). About 20% of the total employment in the EU shipping industry is shore based, while the remaining 80% is based at sea.

Besides the transport of commodities within EU territories and overseas, the maritime transport sector is also vital for transporting passengers (residents and visitors) and for maintaining the connections between different countries or between remote islands and the mainland. Therefore, there is an important social dimension of maritime services. The islands under consideration in the present study currently report about 40 million passengers per year, including tourists and permanent residents (Eurostat 2020). In addition, according to pre-COVID-19 estimations, the Mediterranean was among the world’s fastest-growing cruise markets, with an actual capacity increase of about 10.2% per year (Cruise Industry News 2018).

The broader Mediterranean region is a climate change hot spot that is warming faster than the global rates (Cramer et al. 2018; Zittis et al. 2022). At the same time, a general decline in total precipitation is evident for most of the region (Cherif et al. 2020). Regional climate projections based on multi-model ensembles indicate further warming by 2100 (Zittis et al. 2019; Cherif et al. 2020). According to these studies, this will be within the range of 1 and 5 °C with respect to the end of the previous century, and is expected to be strongest during summer. A general drying (of between 10 and 40%) is also inferred for the Mediterranean, particularly under high-forcing scenarios. For the Canary Islands, warming and drying of a similar magnitude are expected by the end of the century (Expósito et al. 2015; Carrillo et al. 2022). Despite the identification of the region as a prominent climate change hot spot, the range of potential impacts on port and ship operations has not, so far, been extensively explored.

Various climate change stressors can affect both the harbor infrastructure and ships en route. For example, ports are strongly impacted by rising sea levels, affecting port facilities and increasing the risk of flooding (e.g., Torres et al. 2021). The global mean sea-level rise has accelerated in the last century and will likely rise by 0.43 to 0.84 m until 2100, depending on the emission scenario (Pörtner et al. 2019). Due to ocean dynamics and the Earth’s gravitational field, there will also be regional differences in sea-level rise of the order of 0.1 m (Asariotis and Benamara 2012). Seaports are generally resilient to sea level rises plus storm surges of less than 1 m, and can operate without significant interruptions by adopting soft adaptation strategies (Christodoulou and Demirel 2017). Maritime transport can also be affected by climate change through the increase in the intensity of extreme weather events, including tropical-like cyclones (Gaertner et al. 2007; Sánchez-Arcilla et al. 2016; González-Alemán et al. 2019; Hochman et al. 2022; Zittis et al. 2021a; Flaounas et al. 2022). According to climate projections, tropical cyclones are expected to change significantly not in frequency but in intensity, due to rising sea-surface temperatures (Pörtner et al. 2019). For example, a doubling of category 4 and 5 hurricanes in the Atlantic is expected by 2100 (Bender et al. 2010). The resulting extreme winds and waves can harm vessels but can also cause damage and flooding of ports, especially in combination with the rising sea level (Hanson and Nicholls 2012). For example, for the second half of the current century and under a business-as-usual pathway, such a combination is expected to cause an up to 66% increase in inoperable hours in northern Spain (Camus et al. 2019). The port processes affected by weather/climate hazards are dock flooding, basin agitation, port siltation, breakwater stability, overtopping, and scouring (Sánchez-Arcilla et al. 2016).

In addition to the biophysical impacts of weather and climate hazards, socio-economic impacts will likely be introduced (Léon et al. 2021). Such impacts can include an increase in users’ risk perception, leading to lower rates of moorings and turnover, increased costs of maintenance of nautical installations and equipment, increased costs of new investment and insurance, carbon tax effects on fossil fuel prices, less turnover from maritime transport activities, and higher disruption costs (Léon et al. 2021). Most of these impacts and their economic value are still under investigation. Although some ports have already started to adapt to climate change (Becker et al. 2012; Ng et al. 2013, 2018), more investment from the public and private sectors is required (Monios and Wilmsmeier 2020). This is particularly relevant for regional ports, such as those located on islands. The estimated costs and effectiveness of adaptation measures depend on the port location and are often difficult to assess. In most cases, the most effective adaptation measure would be to improve the flood resistance of the transport infrastructure and increase the heights of vulnerable ports (Yang et al. 2018).

**Fig. 1:**
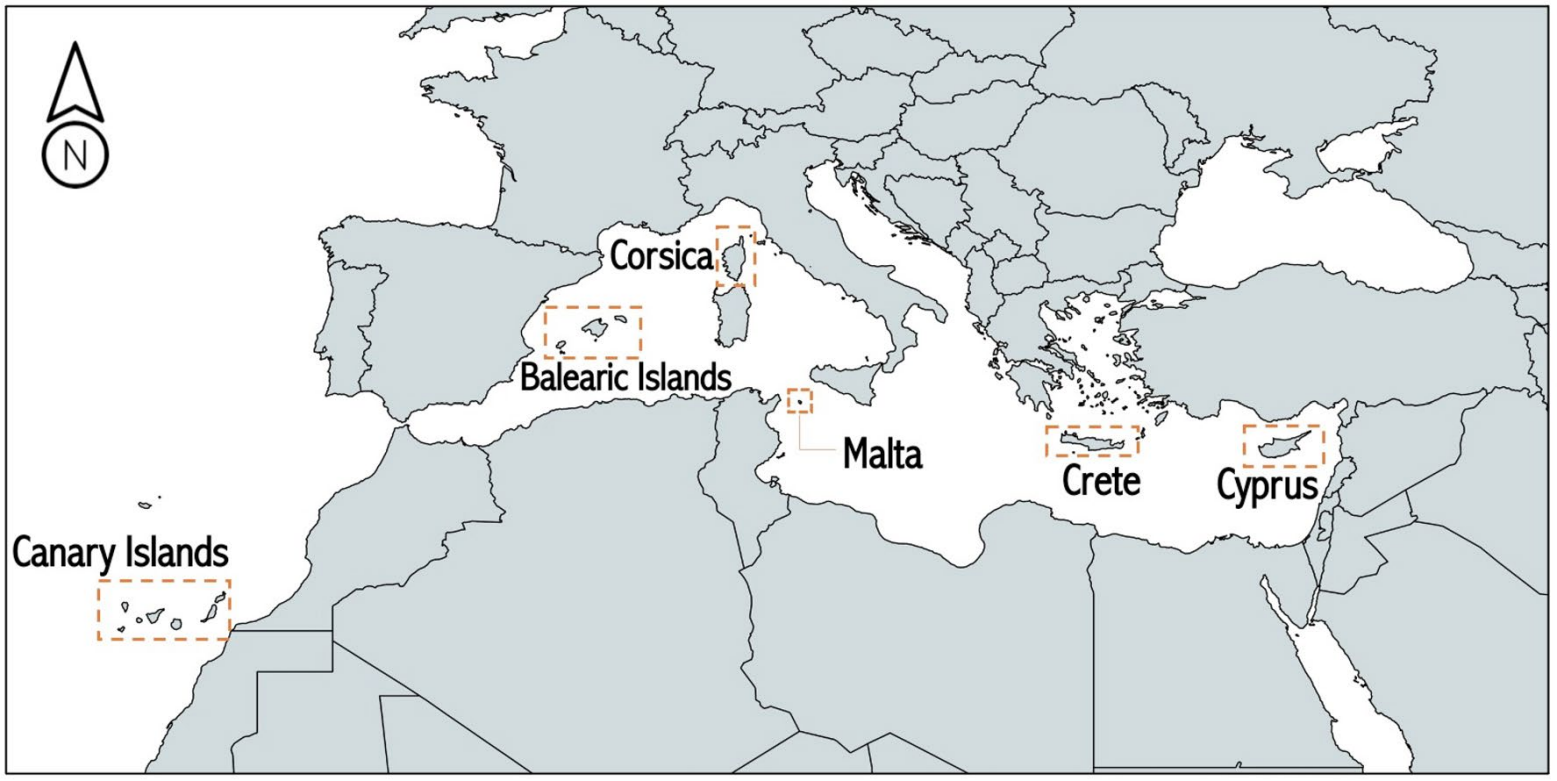
Locations of the six islands and archipelagos under investigation (from west to east: Canary Islands, Balearic Islands, Corsica, Malta, Crete and Cyprus)

Considering the importance of the sector and the lack of targeted impact studies in the broader Euro-Mediterranean region, our main objective is to provide a comprehensive framework to assess the risk of maritime transport disruption under present and future climate change conditions. The widely used impact chain approach is optimized and applied to six European islands or archipelagos that strongly rely on maritime means for transporting passengers and goods and are also common stops for leisure cruise ships. We also aim to decompose the different risk components and better understand the factors that drive future changes. Our framework is supported by state-of-the-art regional climate information and up-to-date socio-economic data. We focus primarily on large islands representative of the Mediterranean environment; nevertheless, European islands in the Atlantic Macaronesia are also considered (Fig. 1). The list of case studies includes the Canary Islands, the Balearic Islands, Corsica, Malta (including Gozo and Comino), Crete and Cyprus; however, our objective is to propose a framework that, with minor adjustments and availability of data, is easily transferred to other locations. The need for timely climate change mitigation efforts is investigated by comparing the results of a ‘business-as-usual’ scenario with a pathway closer to meeting the Paris Accord’s main targets (i.e., keeping global warming to less than 2 °C since the pre-industrial era). Finally, based on our results, we complement the discussion by proposing targeted adaptation measures for reducing the climate-driven risk of maritime transport disruption in the region of interest.

## Data and methods

### Climate data

An important component of the present analysis is the climatic data used for the calculation of the hazard indicators. These include parameters such as the sea-level rise and extreme winds and waves for historical and future climate projections. Near-surface wind data were obtained from a large ensemble of regional climate simulations performed under the Coordinated Regional Downscaling Experiment (CORDEX) (Giorgi and Gutowski 2016; Diez-Sierra et al. 2022). Information integrated for the European (EURO-CORDEX) and the Middle East and North Africa (MENA-CORDEX) domains of CORDEX was used for the Mediterranean and Atlantic regions, respectively, at horizontal resolutions of 0.11° (≈ 12 km) and 0.44° (≈ 50 km). More information on the simulations and domain extents can be found in Jacob et al. (2020), Obermann-Hellhund et al. (2018), and Zittis et al. (2021b). Extreme wave projections for the Mediterranean were available from the Med-CORDEX initiative at a horizontal resolution of 0.11° (Ruti et al. 2016; Soto-Navarro et al. 2020). However, for the Canary Islands, located in the Atlantic Ocean, additional wave simulations were performed using the WAVEWATCH III model (WW3DG 2016), driven by meteorological input from the Hadley Centre Global Environmental Model (HadGEM). WAVEWATCH III solves the random-phase spectral action density balance equation for wavenumber-direction spectra. These simulations were performed at a horizontal resolution of 0.25°, covering a domain from 10 to 42°N and 70 to 5°W.

### The impact chain approach

Impact chains (ICs) are an effective way to visually synthesize the complex relationships between Exposure (to mean climate conditions or hazards), Sensitivity (related to physical and socio-economic features) and Adaptive Capacity of the system under investigation. In more detail, an impact chain is an analytical tool that helps us to better understand, systemize and prioritize the factors that drive vulnerability, and thus risk, in a system under review (GIZ 2014). This could be either a human or natural system. The concept of ICs was introduced by Schneiderbauer et al. (2013) and was refined by the German Cooperation for International Cooperation (GIZ) in their Vulnerability Sourcebook (GIZ 2014). Impact chains have since become more and more widely used as a climate risk assessment method at the regional-to-local level for research and decision-making support. Successful examples include the assessment of climate change impacts on several socio-economic sectors, including agriculture, water and land resource management, tourism, as well as ecosystem-based adaptation (Hagenlocher et al. 2018; Arabadzhyan et al. 2020; Schneiderbauer et al. 2020; Léon et al. 2021; Zebisch et al. 2021).

**Fig. 2:**
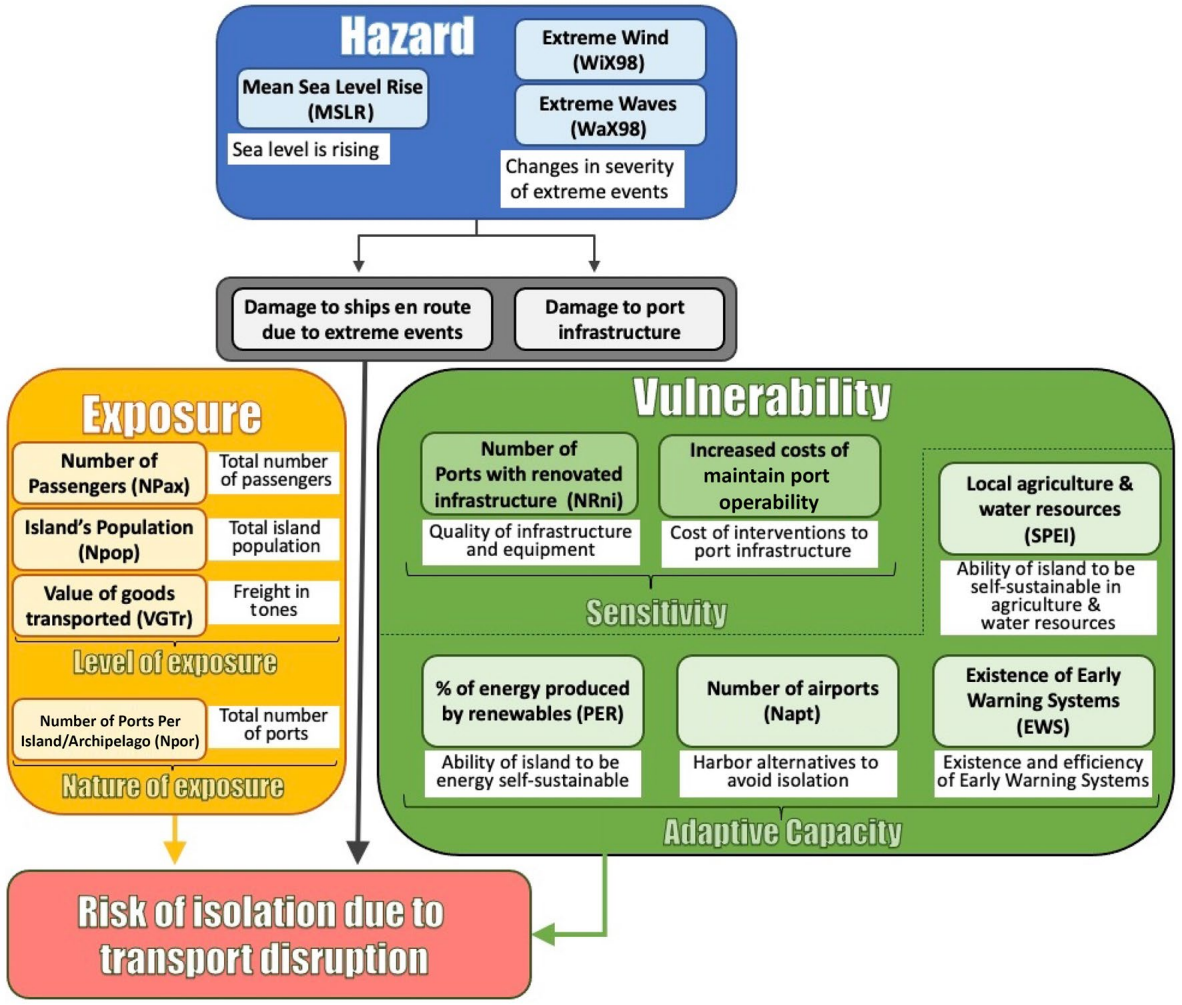
Conceptualization framework for the impact chain operationalization for the risk of isolation due to maritime transport disruption

The methodology can be used for both the high-level identification of key risks as well as a more in-depth analysis of specific risks and adaptation strategies. Some of the advantages of using this framework include its flexibility and simplicity in terms of calculations, its applicability to different scales (from national to local), and the consideration of the entire planning cycle of the adaptation process (Zebisch et al. 2021). This can range from identifying the adaptation demand to selecting measures to monitor and evaluate the success of adaptation interventions in lowering vulnerability. Several variations of ICs have been proposed; however, in the present study, we apply the general framework presented by Zebisch et al. (2017) and Arabadzhyan et al. (2020), which makes the assessment approach with impact chains compatible with the concept of risk used in the Fifth Assessment Report of the Intergovernmental Panel on Climate Change (IPCC 2013). This conceptualization framework was adjusted for the risk of isolation due to maritime transport disruption (Fig. 2) and was operationalized for the six EU islands and archipelagos under investigation (the Canary Islands, the Balearic Islands, Corsica, Malta, Crete and Cyprus). Three main components drive risk:

(i) Hazard: related to meteorological conditions, extreme weather and changes in such physical phenomena due to global warming.
(ii) Exposure: related to the presence of people, livelihoods, services, infrastructure, and economic, social or other assets.
(iii) Vulnerability: related to the propensity or predisposition to be adversely affected. Vulnerability encompasses a variety of elements, including Sensitivity (i.e., susceptibility to harm) and Adaptive Capacity (i.e., the capacity to cope and adapt).

### Description of indicators and scenarios

As depicted in Fig. 2, several indicators have been identified for each risk component. For climate change-driven hazards, we considered the regional mean sea-level rise (MSLR), extreme waves (WaX98) and extreme wind (WiX98). The two extreme weather indicators (WaX98 and WiX98) were extracted from the 98^th^ percentile of daily maximum values for each year. Exposure indicators include the number of passengers (NPax), the island's total population (Npop), the value of transported goods expressed as the total annual freight (VGTr) and the number of ports per island or archipelago (Npor). Indicators for sensitivity include the quality of the port infrastructure (Nrni) and the increased cost of keeping ports operable (ICost). Finally, for the Adaptive Capacity component, the proposed indicators are the percentage contribution of renewables to energy production (PER), the existence and efficiency of early warning systems (EWS), harbor alternatives such as airports (Napt), and the standardized precipitation evaporation Index (SPEI) as a proxy for island sustainability in water resources and agriculture. Since the risk is reduced when the Adaptive Capacity is high, indicators of this component were treated inversely, i.e., high values were assigned low scores. A more detailed description of indicators is presented in Supplementary Appendix A.

Besides the historical reference period, we considered two 20-year future periods: one near the middle of the twenty-first century (2046–2065) and one covering the end of the twenty-first century (2081–2100). Therefore, when these were available, we considered projections or estimations for the indicators to assess future risk. This was mainly the case for the components of hazard (mean sea level rise, extreme waves and wind), exposure (population, number of passengers, value of goods), the contribution of renewables, and the potential for sustainable water resources and agriculture. Two representative concentration pathways (RCPs) were considered for meteorological hazards (Meinshausen et al. 2011). One “high-emission” or “business-as-usual” pathway (RCP8.5) and a more optimistic one (RCP2.6) that is closer to the main targets of the Paris Accord to keep global warming to lower levels than 2 °C since pre-industrial times. Regarding future estimations of exposure indicators, we scaled the observed values according to the population projections (for the years 2050 and 2090). These projections were derived from the United Nations Department of Economic and Social Affairs (https://population.un.org/wpp/).

### Normalization of indicators, weighting and risk calculation

Prior to the calculation of risk, the indicators needed to be normalized to values between 0 and 1. For this, we have applied the minimum–maximum normalization method as described in OECD (2008) and GIZ (2014). The methodology is defined in the following formula:

1 $$X_{i, 0\; to \;1} = \frac{{X_{i} - X_{\text{MIN}} }}{{X_{\text{MAX}} - X_{\text{MIN}} }},$$

where *X*_*i*_ represents the individual data point to be transformed, *X*_MIN_ is the lowest value for that indicator, *X*_MAX_ is the highest value for that indicator, and *X*_*i*, 0 to 1_ is the new value to be calculated (i.e., the normalized data point within the range of 0 to 1). For most of the exposure and vulnerability components, this normalization was applied across the different islands in order to facilitate an inter-island comparison and to prioritize the cases of higher risk. As an example, for the Npop indicator,* X*_MIN_ is the value for the island with the lowest population (Corsica) and *X*_MAX_ is the value for the archipelago with the highest population (Canary Islands). Therefore, we assigned a value of 0 for the former and a value of 1 for the latter, while the rest of the islands were assigned values in between. For the extreme hazard indicators, in order to provide normalized values that are meaningful in terms of physical impacts, we have set the minimum/maximum values according to expert judgement. Critical thresholds of the Beaufort and Douglas scales (Owens 1982) were used for extreme winds and waves, respectively. Values before and after the normalization, as well as more information on the sources of each indicator, are presented in Supplementary Appendix A.

Regarding the weighting of the different risk components, several weights have been examined; however, based on expert judgement (in-depth interviews with experts and stakeholders were conducted for each island under study). The number and expertise of stakeholders were different on each island. In particular, we involved port managers, maritime transport experts, oceanographers, climatologists and other experts. More information on the process, which involved the organization of local working groups and online survey tools, is available at https://soclimpact.net/reports/. To achieve a more subjective analysis and facilitate an impartial island inter-comparison, we conservatively assigned equal weights to all the main components of risk (i.e., 0.33 to Hazard, 0.33 to Exposure and 0.33 to Vulnerability). For the sub-components of Exposure (see Fig. 1), we have assigned a weight of 0.33 to the Nature of Exposure and a weight of 0.66 to the Level of Exposure, since the latter is of greater importance. Similarly, for the sub-components of Vulnerability, we have assigned a weight of 0.33 to the factors of sensitivity and a weight of 0.66 to the factors of Adaptive Capacity. The selection of weights is a subjective decision; nevertheless, we consider our selection to be quite conservative, and therefore we believe that a slightly different choice would not significantly affect our calculations.

Finally, after the normalization of indicators and the application of weights to the different components, the relative risk for maritime transport disruption is calculated according to the following formula:

2 $$\begin{gathered} {\text{Risk}} = \left( {0.33 \times {\text{Hazard}}} \right) + \left( {0.33 \times \left( {0.33 \times {\text{Nature}}\,{\text{of}}\,{\text{Exposure}} + 0.66 \times {\text{Level}}\,{\text{of}}\,{\text{Exposure}}} \right)} \right) \hfill \\ \;\;\;\;\;\;\,\;\;\;\;\; + \left( {0.33 \times \left( {0.33 \times {\text{Factors}}\,{\text{of}}\,{\text{Sensitivity}} + 0.66 \times {\text{Factors}}\,{\text{of}}\,{\text{Adaptive}}\,{\text{Capacity}}} \right)} \right). \hfill \\ \end{gathered}$$

The derived relative risk values calculated for each island and period of analysis were eventually categorized into five classes for better visualization and interpretation of the results. Values between 0 and 0.2 indicate a very low risk, values between 0.2 and 0.4 indicate a low risk, values between 0.4 and 0.6 indicate a medium risk, values between 0.6 and 0.8 indicate a high risk, while greater values indicate a very high risk for maritime transport disruption.

## Results

### Overview for all islands

For the recent-past/present conditions, the operationalization of the maritime transport impact chain indicates a low risk for all investigated islands (Table 1). In general, the maritime transport sectors of the larger islands (e.g., Corsica, Cyprus and Crete) are more resilient to the impacts of climate change. Up to a point, this is related to a large number of harbor alternatives compared to smaller islands. Results for the future highlight the importance of adopting a low-emission pathway, since this will keep the risk for maritime transport disruption similar to present conditions, while for some islands the risk is expected to decline slightly.

**Table 1:** Historical (1986–2005) and future risks of isolation due to maritime transport disruption for six European islands and archipelagos, as calculated based on the impact chain approach (0 = low risk, 1 = high risk)

	Historical reference	RCP2.6 (2046–2065)	RCP2.6 (2081–2100)	RCP8.5 (2046–2050)	RCP8.5 (2081–2100)
Cyprus	0.21	0.21	0.23	0.28	0.33
Crete	0.19	0.20	0.21	0.26	0.30
Malta	0.33	0.33	0.34	0.40	0.44
Corsica	0.19	0.19	0.20	0.24	0.29
Canary Islands	0.32	0.33	0.30	0.41	0.44
Balearic Islands	0.28	0.28	0.28	0.33	0.38

**Fig. 3:**
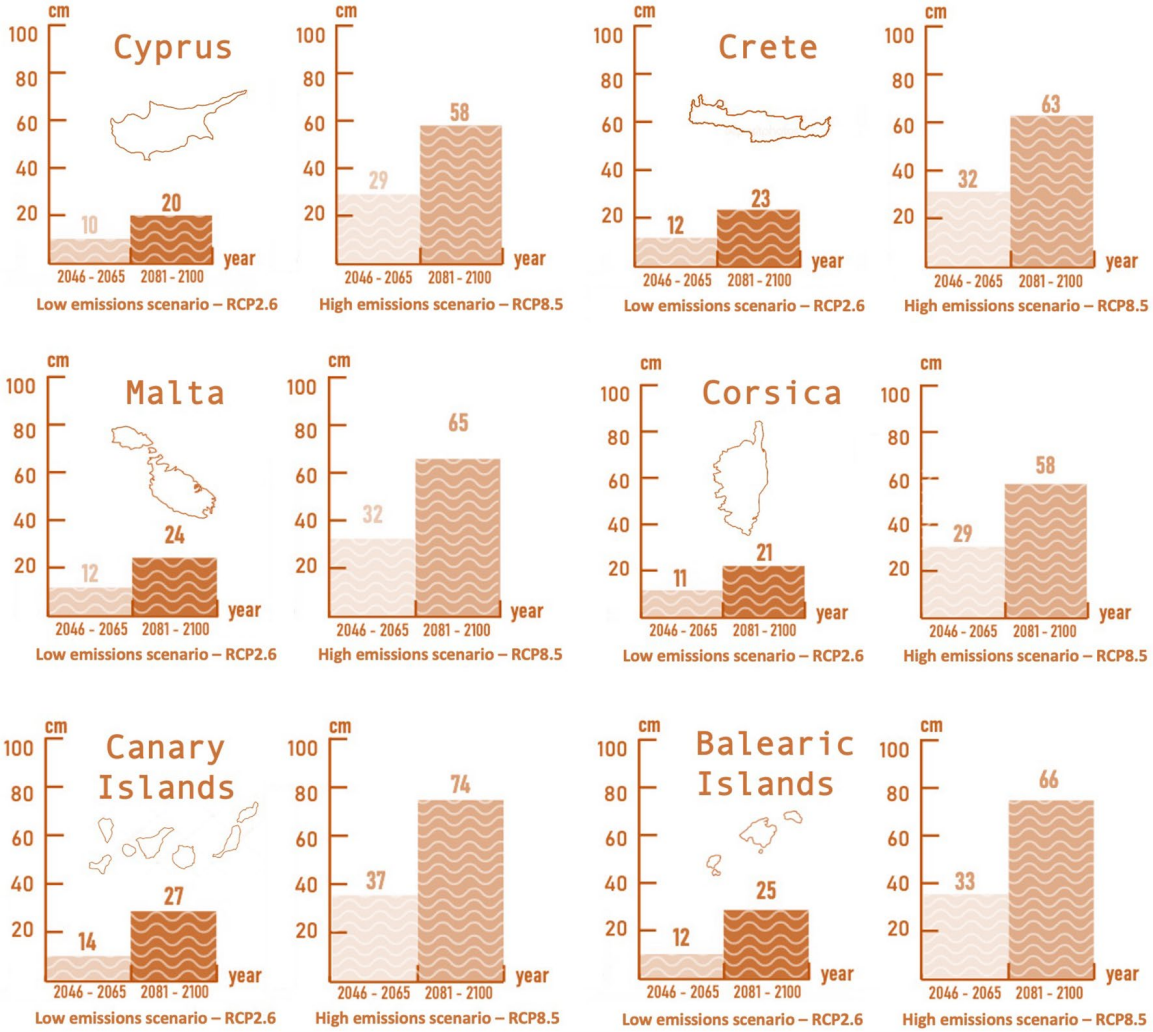
Projected mean sea level rises with respect to the 1986–2005 reference period for six European islands and archipelagos under two future emission pathways

Concerning changes in climate hazards, these are more important when it comes to the regional mean sea-level rise. A visual summary of the mean sea-level rises for all islands with different horizons and scenarios is presented in Fig. 3. For RCP2.6, the mean sea-level rise is not expected to exceed 27 cm with respect to the historical reference. On the contrary, a business-as-usual pathway (RCP8.5) implies an up to three times higher mean sea-level rise for both periods. Particularly at the end of the current century, the mean sea level rise will likely reach 58 cm in all islands, while in the Canary Islands, this is projected to reach 74 cm—the greatest increase among all the case studies. On the contrary, the projected changes are not expected to be significant for extreme wind and waves (Supplementary Tables A2–A5).

In the inter-island comparison, Malta’s maritime sector is found to be the most vulnerable; nevertheless, future risk, even under the high-emission pathway (RCP8.5), is not expected to exceed medium risk values. In contrast, Corsica is the island least susceptible to climate change impacts. Detailed tables of the impact chain operationalization (including all components, islands and future scenarios) are presented in Supplementary Tables B.1–B.5 of Supplementary Appendix B. More detailed results for each investigated island are presented in the following six subsections.

### Cyprus

**Fig. 4:**
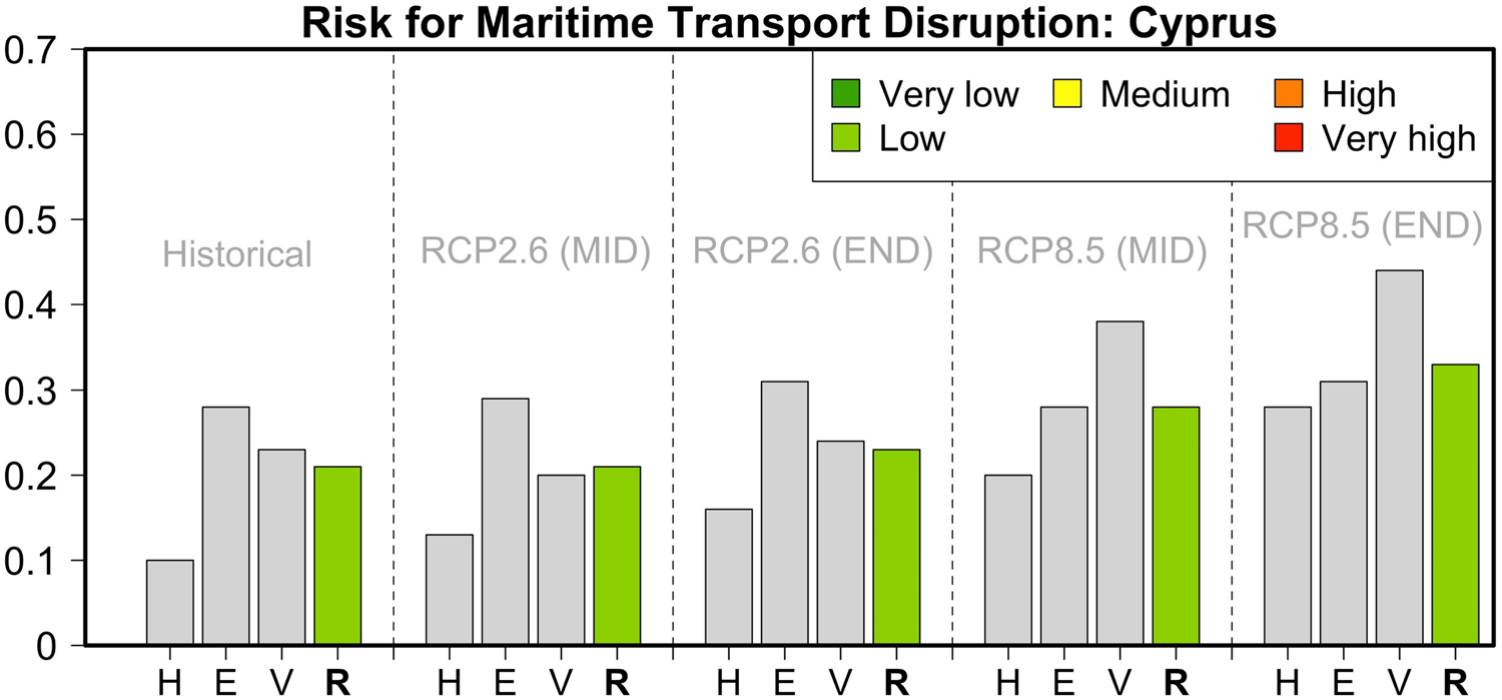
Hazard (*H*), Exposure (*E*) and Vulnerability (*V*) components and relative risk (*R*) values for the risk of maritime transport disruption in Cyprus during the historical reference period and two future periods under pathways RCP2.6 and RCP8.5

For the eastern Mediterranean island of Cyprus, our analysis indicated that the value for the risk of isolation due to maritime transport disruption was low (0.21) during the historical reference period (Table 1 and Fig. 4). The greatest contributions came from the socio-economic drivers of Adaptive Capacity and Nature of Exposure. On the contrary, the indicators related to meteorological hazards (sea level rise, extreme winds and waves) had a much smaller contribution. For the mid-century, the risk for transport disruption remains low for both the RCP2.6 and RCP8.5 pathways (values of 0.22 and 0.23, respectively).

The contribution of Hazard indicators is expected to become more significant, since the mean sea-level rise is increased compared to the default zero value of the historical reference period. Since the Exposure indicators have the same values for both pathways, the differences in the risk values for this period are mainly driven by the factors of Adaptive Capacity, and primarily the contribution of renewables to the total energy production and the ability to be self-sustained in terms of water resources and agriculture. These indicators of sustainability are more advantageous in an RCP2.6 future. As a result, the risk values are somehow lower under this pathway. By the end of the twenty-first century, the risk values are not projected to change much for the optimistic RCP2.6 pathway. On the contrary, for RCP8.5, our analysis indicates a further increase in the risk value (0.33). Nevertheless, due to the lower contributions from the Exposure and Vulnerability components, the future risk for maritime transport disruption in Cyprus is still categorized in the low-risk class.

### Crete

**Fig. 5:**
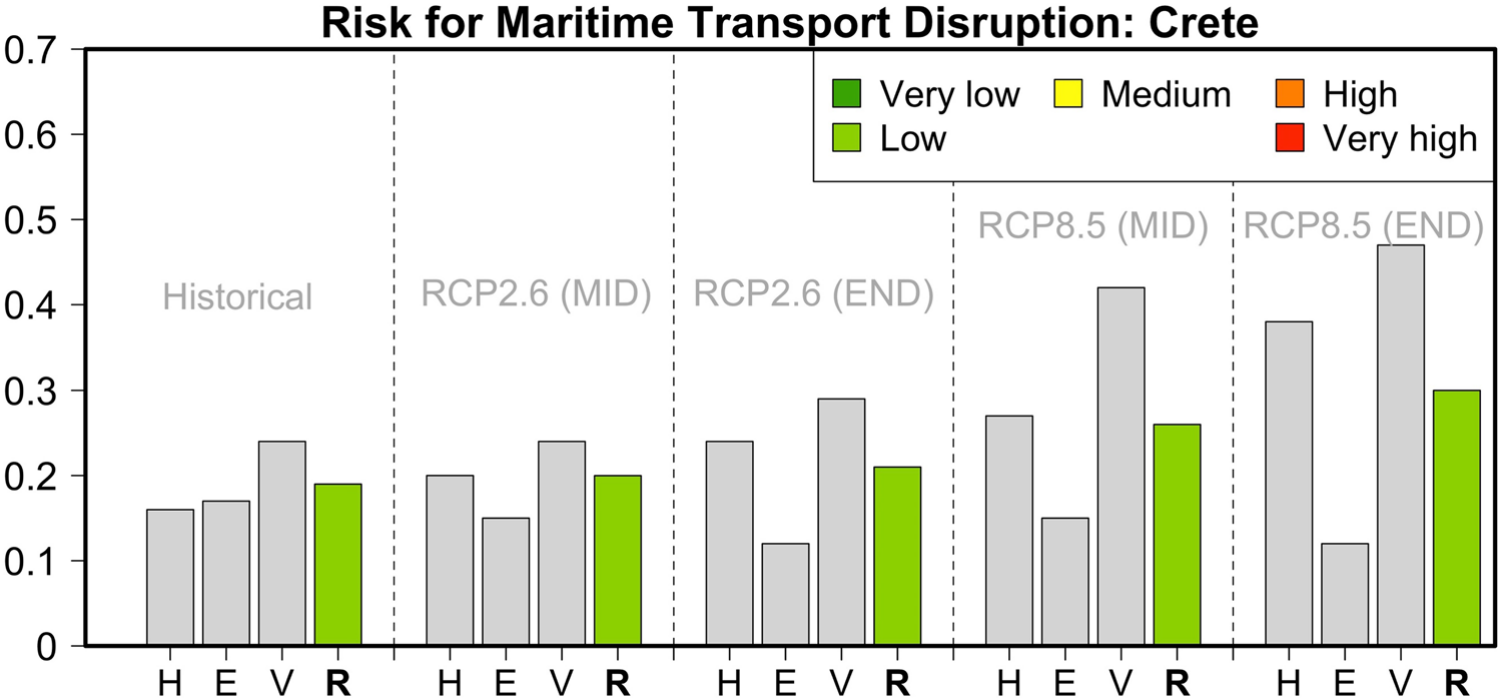
Hazard (*H*), Exposure (*E*) and Vulnerability (*V*) components and relative risk (*R*) values for the risk of maritime transport disruption in Crete during the historical reference period and two future periods under pathways RCP2.6 and RCP8.5

For the largest Greek island, during the historical reference period, the IC operationalization indicates similar conclusions can be drawn to those for the case of Cyprus (Table 1 and Fig. 5). The risk value is characterized as one of the lowest (0.19), with the most significant contribution arriving from the factors of Adaptive Capacity. This is due to the low contribution of renewables and the relatively low number of harbor alternatives (e.g., airports) on this particular island. For RCP2.6, the risk of transport disruption is projected to increase in both time horizons. Despite a higher contribution of renewable energy, access to maritime transport alternatives will be reduced since this pathway also implies a 10–40% reduction in aviation activities (see Supplementary Appendix A). Nevertheless, the increased contribution of renewables remains important since it makes the island less dependent on imported fossil fuel for energy production and therefore increases its capacity to adapt and be self-sustained. For the business-as-usual pathway RCP8.5, our analysis indicates an increase for the end of the current century (a risk value of 0.30). This increase can be attributed to the projected augmentation of meteorological hazards (mainly extreme winds and the mean sea-level rise). In addition, the transition to warmer and drier conditions (high SPEI values) indicates a lower capacity to adapt when it comes to sustainability in water resources and local food production. The fact that Crete is one of the islands where the levels of Exposure indicators (population, number of passengers, and value of goods) are expected to decrease substantially keeps the future risk for transport disruption at relatively low levels.

### Malta

**Fig. 6:**
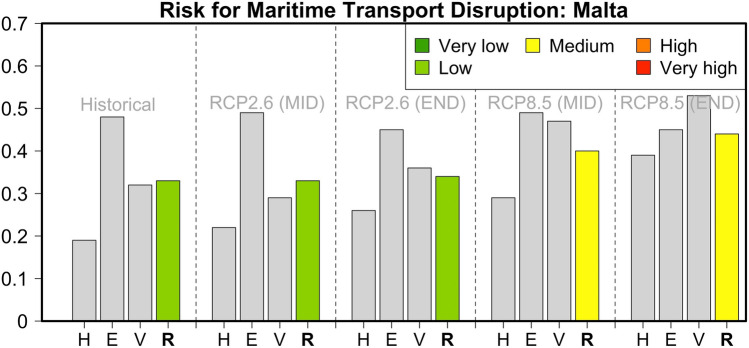
Hazard (*H*), Exposure (*E*) and Vulnerability (*V*) components and relative risk (*R*) values for the risk of maritime transport disruption in Malta during the historical reference period and two future periods under pathways RCP2.6 and RCP8.5

Compared to Cyprus and Crete, the IC operationalization for Malta (Fig. 6) reveals a higher present relative risk for isolation due to maritime transport disruption (a risk value of 0.33). This is mainly related to the high importance of Exposure indicators (Nature and Level of Exposure), which is the combined result of the small number of ports and the high value of goods expressed as the total freight. Two other contributors to the relatively high risk value are increased Vulnerability due to the small number of harbor alternatives (i.e., airports and aviation activity) and the small percentage of renewables in total energy production. For future decarbonization pathway RCP2.6, the risk is expected to remain at similar levels, mainly because an anticipated decrease in aviation activities is counterbalanced by an increase in the renewable energy contribution. On the contrary, under the RCP8.5 pathway, the risk for transport disruption in the Maltese islands is projected to increase to medium values (0.40–0.44). This is due to the lower contribution of renewables in this high-emission scenario and the projected increase in Hazard indicators (mainly extreme winds and the mean sea level rise). In particular, the mean sea level in the region is expected to rise by 65 cm, thus posing an additional threat to harbor infrastructure. In addition, the island's potential for sustainability in terms of water resources and agriculture will be significantly lower. At the end of the century, the Exposure indicators (e.g., the number of passengers, the value of goods) are considered to be decreased in both scenarios.

### Corsica

**Fig. 7:**
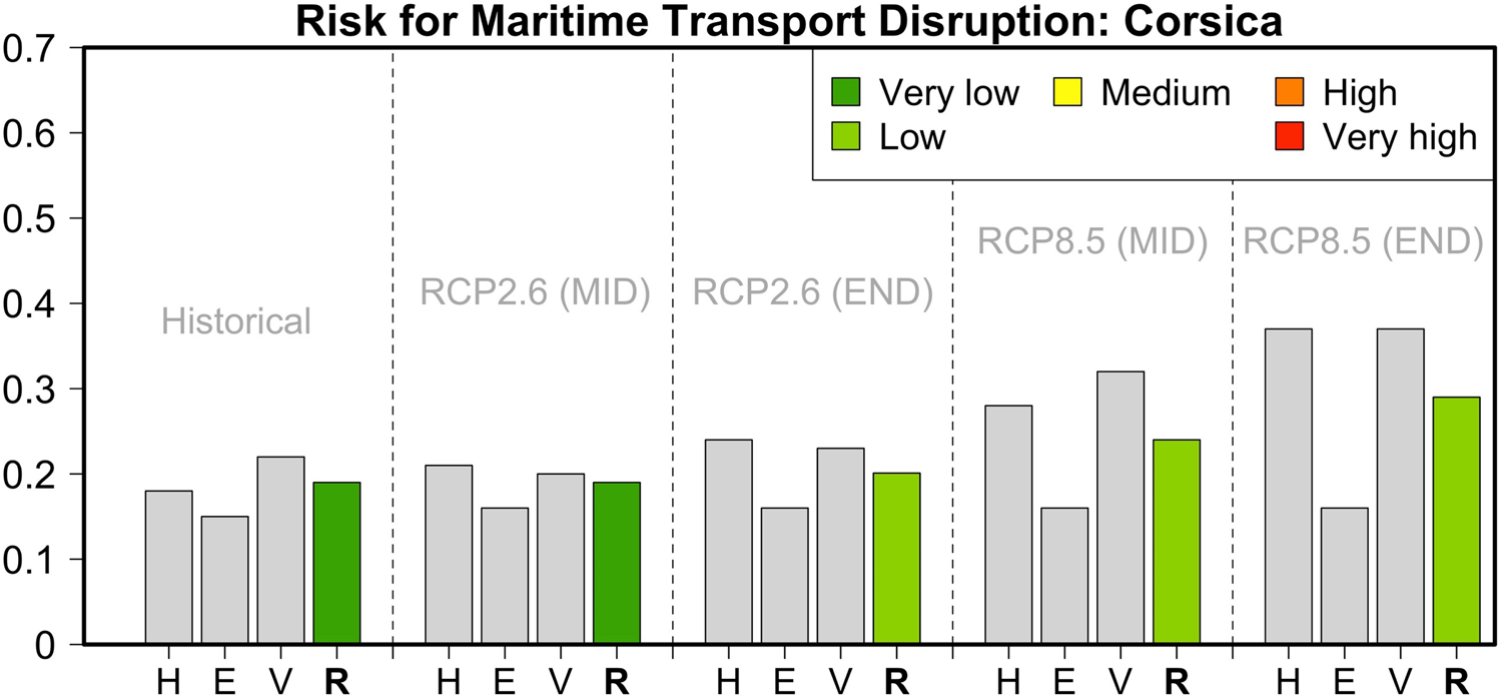
Hazard (*H*), Exposure (*E*) and Vulnerability (*V*) components and relative risk (*R*) values for the risk of maritime transport disruption in Corsica during the historical reference period and two future periods under pathways RCP2.6 and RCP8.5

The maritime transport sector on the island of Corsica in France is found to be less susceptible to climate change, as our impact chain operationalization indicates that it has the lowest risk value among all the investigated islands (a risk value of 0.19 for the present conditions). This is similar to the case for Crete in Greece. This low risk value is mostly related to the low contribution of the Exposure indicators (for example, the number of passengers, the value of transported commodities, etc.). Under pathway RCP2.6, the relative risk value will likely remain low because an increase in the Adaptive Capacity component counterbalances the adverse effect of increasing meteorological hazards. This is mostly driven by the fact that the percentage of renewables is expected to increase in this decarbonization pathway (Fig. 7). Under the business-as-usual pathway RCP8.5, and mainly due to an increased contribution from future meteorological hazards, the risk is expected to increase slightly by the mid-twenty-first century (a risk value of 0.24) and to reach a value of 0.29 by 2100. The future transition to drier and warmer conditions also adversely impacts Corsica’s potential for sustainability in terms of water resources and agriculture.

### Canary Islands

**Fig. 8:**
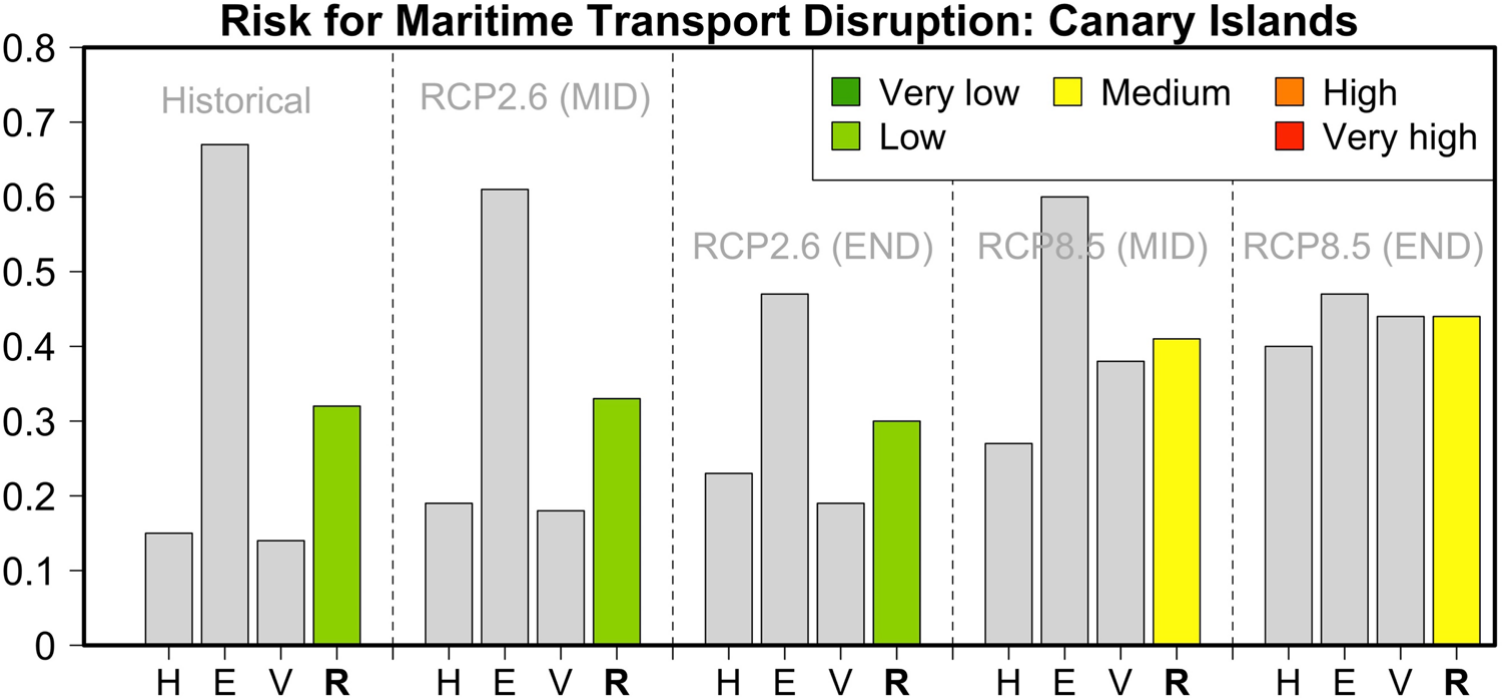
Hazard (*H*), Exposure (*E*) and Vulnerability (*V*) components and relative risk (*R*) values for the risk of maritime transport disruption in the Canary Islands during the historical reference period and two future periods under pathways RCP2.6 and RCP8.5

The Canary Islands are the focus of our only case study of an archipelago outside the Mediterranean. For the historical reference period, our analysis determined a low risk value of 0.32 (Fig. 8). That is the second-largest risk value overall after Malta. This result is clearly due to the contribution of Exposure indicators. In particular, the total population, the number of passengers, and the value of goods are the highest among all the investigated islands. Under an RCP2.6 sustainability pathway, the risk value is expected to remain stable or decrease marginally (risk values of 0.33 and 0.30 for the middle and end of the twenty-first century). This is mainly due to the combination of increased Adaptive Capacity and a reduced contribution from Exposure indicators, since the archipelago’s population was assumed to be declining (i.e., following the trends for mainland Spain). Under pathway RCP8.5, this decrease in the Exposure components is counterbalanced by a significantly increased contribution of meteorological hazards due to stronger climate change effects. As the Canaries are located in the Atlantic Ocean, the predicted mean sea-level rise (0.74 cm) is the highest among all the investigated islands or archipelagos. In addition, the Adaptive Capacity contribution will be higher compared to the decarbonization pathway RCP2.6, mainly due to the impact of increased SPEI values, which indicate a lower potential for agro-food sustainability. As a result of these synergies, the end-of-the-century risk value will be increased to medium levels (0.44). Together with Malta, this is the highest risk value among the islands assessed.

### Balearic Islands

**Fig. 9:**
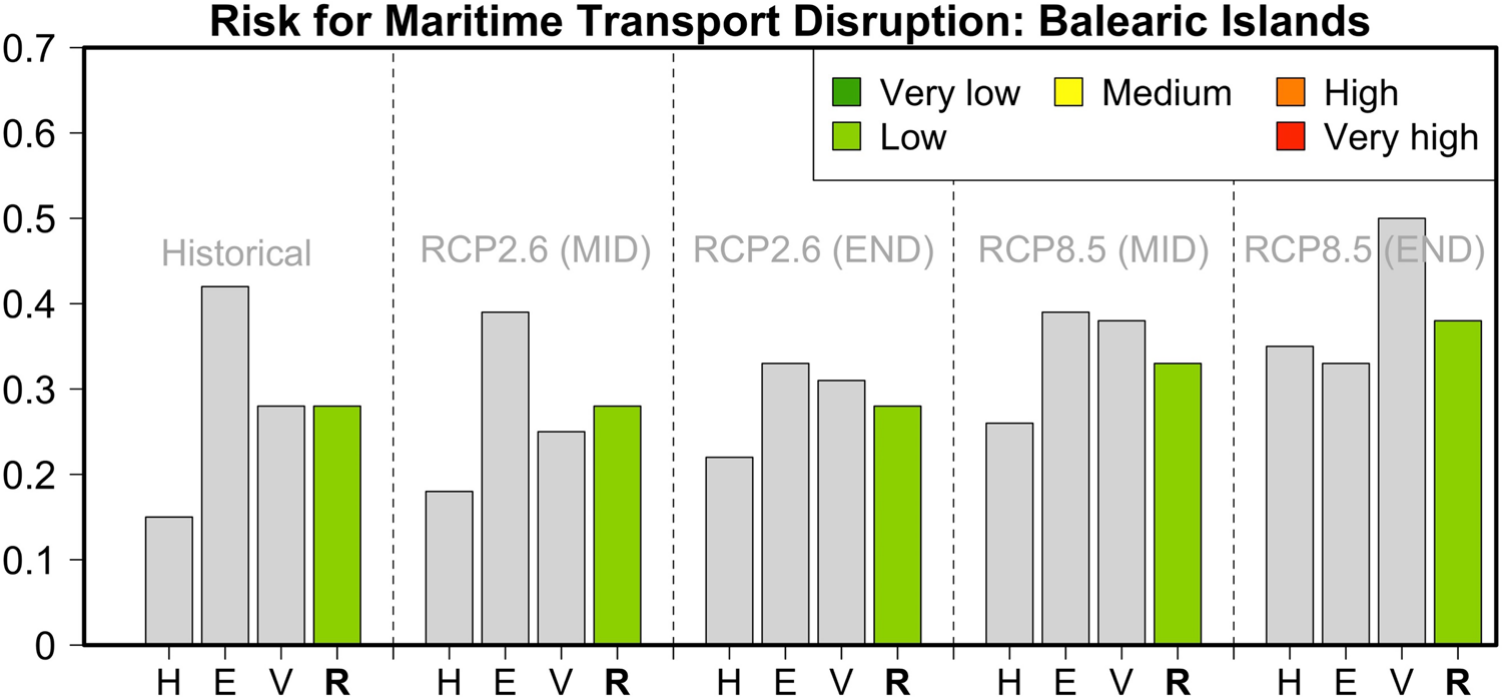
Hazard (*H*), Exposure (*E*) and Vulnerability (*V*) components and relative risk (*R*) values for the risk of maritime transport disruption in the Balearic Islands during the historical reference period and two future periods under pathways RCP2.6 and RCP8.5

The Balearic Islands in the western part of the Mediterranean are the last archipelago investigated for the impact of climate change in our assessment. For the historical reference period, the impact change operationalization resulted in a risk value of 0.28 (low risk), corresponding to the third-highest risk overall (Fig. 9). The greatest contribution to the overall risk comes from the low Adaptive Capacity because of the small number of harbor alternatives and the low percentage of renewables for energy production on the island. Since the contribution of renewables is expected to increase under RCP2.6 whereas the contribution of Exposure and Hazard indicators will likely not significantly change in the coming decades, the risk under this scenario remains at levels similar to the reference period. For the business-as-usual RCP8.5, the risk is anticipated to increase (to values of 0.33–0.38) as a result of the meteorological hazards (mainly extreme winds and the mean sea-level rise), a lower contribution of renewable energy production, and a lower potential for sustainable water resources and agriculture, which translates into a greater need for food imports.

## Conclusions and discussion

We conceived a comprehensive framework, defined and determined the relevant indicator values, and applied it for the assessment of the historical and future risk of maritime transport disruption in six European islands and archipelagos. Our results, based on state-of-art regional climate modeling data and local information on socio-economic drivers, highlight that for all of the investigated case studies, the future risk is not expected to exceed medium values. This conclusion is in agreement with previous studies (e.g., Izaguirre et al. 2020). The highest risk values are found for the islands with a limited number of harbor alternatives (as in the case of Malta) or high levels of exposure components (for instance, the Canary Islands). According to the updated regional projections used, climate change in the regions under investigation is expected to exacerbate the future risk mainly through the increased mean sea levels and, to a lesser extent, from changes in wind and wave extremes. In addition, the projected warming and drying, particularly under the business-as-usual pathway (RCP8.5), will lower the potential for sustainable water resources and agriculture and increase the demand for food imports. Nevertheless, for some islands, this increase in particular hazard components is counteracted by a projected decrease in the population (and thus in the number of passengers and value of goods), and, therefore, future risk values will likely remain at low-to-medium levels.

The present results can support policymakers and stakeholders in identifying and decomposing the major drivers of current and future risk for maritime transport disruption, where the drivers considered are weather and climate hazards. For most islands under investigation, the current and near-term future risk is mostly driven by the Vulnerability and Exposure components rather than the natural hazards per se. The latter are found to play a more important role towards the end of the current century and under the business-as-usual pathway. Therefore, our analysis corroborates that adopting timely and aggressive mitigation measures through low greenhouse gas emission and concentration pathways (e.g., RCP2.6) will keep the risk for maritime transport disruption similar to present-day levels. For some islands, when assessed in combination with demographic and socio-economic data projections, the risk is even expected to decline slightly (up to 10%). On the contrary, a business-as-usual pathway (e.g., RCP8.5) leads to an increased risk for isolation due to maritime transport disruption. This is projected to be higher for all cases towards the end of the twenty-first century.

According to our findings, the severity of extreme winds and waves that could cause damage or disruptions to ships en route is not expected to alter significantly. Since the most relevant future hazard for maritime transport disruption is the mean sea-level rise, adaptation strategies need to focus more on seaport infrastructure. Although some ports have already begun to adapt to climate change conditions, the development and implementation of additional adaptation strategies and measures are strongly recommended to secure the seamless operation of the maritime transport sector. Such interventions can include (a) increasing the number of port alternatives and the establishment of backup routes during and after extreme weather events, (b) the enhancement of early warning systems, (c) the construction or expansion of coastal defences, (d) interventions to increase the height of critical infrastructure, or (e) the reinforcement of inspections, repairs, and the maintenance of port infrastructure and vessels (Becker et al. 2018; Léon et al. 2021). For effective adaptation, port capital-facilities planning should be expanded beyond the 5- to 10-year horizon, which is the current practice of most port operators (Becker et al. 2012, 2018). Moreover, the cost of such adaptation measures can exceed several million euros per year and strongly depends on the future emission pathway and associated hazards. For example, for the selected islands, the operational cost of increasing the height of critical port infrastructure can exceed 15 million euros per year and is about three times higher for the business-as-usual pathway RCP8.5 compared to RCP2.6 (Léon et al. 2021). Port relocation could be an option but only where higher inundation levels (e.g., exceeding 3 m) occur and depending on the importance of the seaport, as this is a very costly solution (Christodoulou and Demirel 2017). According to our estimations, this is typically not a feasible option for the investigated islands.

The proposed framework mainly considered the direct impacts of climate change on the maritime transport of goods and passengers. For example, we did not specify the effect of such disruptions in the supply chain and the value of commodities and services. For the examined islands, a second-level analysis of the indirect effects and linkages between additional socio-economic sectors (e.g., energy and tourism) is presented in Vrontisi et al. (2022). Based on the same climate hazard projections as the present study, their assessment quantified the cumulative losses in gross domestic product (GDP) in various Blue Economy sectors. For maritime transport, the projected GDP losses over the 2040–2100 period can reach 3% (e.g., for the Canary Islands under a business-as-usual pathway).

Parts of the methodology and analysis were inevitably based on experts’ opinions and, therefore, subjective decisions (e.g., the selection of indicators, the normalization of data, and the weighting of the risk components); however, we consider our choices to be conservative and, therefore, we believe that slightly different values would not have affected the main conclusions drawn. For example, our regional projections for Mediterranean sea-level rises are in the lower range of the IPCC Working Group I Interactive Atlas (Iturbide et al. 2021). Similarly, the population projections (also used for scaling some of the exposure indicators) are within the range of projections defined by the latest shared socioeconomic pathways (O’Neill et al. 2017). With minor adjustments, and if reliable climate and socio-economic data are available, the proposed impact chain framework can be applied in other regions or sectors to support policy and decision-making accordingly.

## Supplementary information

Below is the link to the electronic supplementary material.

Supplementary file1 (DOCX 479 KB)

Supplementary file2 (DOCX 508 KB)

## Data Availability

Sources of data on exposure and vulnerability indicators are provided in the Supplementary Annexes. The climate hazard indicators are available in https://zenodo.org/communities/soclimpact. Raw climate data are available upon request.

## Footnotes

1 https://ec.europa.eu/transport/modes/maritime/maritime-transport_en
