# Supplementary material for: Maritime transport and regional climate change impacts in large EU islands and archipelagos
Source: EuroMediterr J Environ Integr. 2023 May 26:1–14. Online ahead of print. doi: 10.1007/s41207-023-00370-6 (PMC10213571; doi:10.1007/s41207-023-00370-6)
Supplement: Supplementary file 1 — Supplementary file1 (DOCX 479 KB) [file 41207_2023_370_MOESM1_ESM.docx]

# **APPENDIX A. Relative Risk Computation I: normalization of sub-component indicators**

##

## A1. Hazard

**Extreme Waves** (**WaX98)**

Extreme waves are defined as the annual 98^th^ percentile of the model-based daily wave height values. The values are averaged for each island under investigation. The units of the WaX98 index are meters. According to the modelled values (provided through CORDEX regional climate simulations), for the 1986-2005 control reference period, WaX98 values range from 1.43 m for Cyprus to 2.27 m for the Canary Islands (Table A.1). We have used the minimum-maximum normalization method. However, since the range of data for the different islands is very small, in order to provide meaningful values after the normalization we have set the minimum/maximum values manually according to expert judgement in a similar way as it is described in OECD (2008) and GIZ (2017). In more detail, a minimum value of 0 is assigned for extreme waves of 1.25 m height and a maximum value of 1 is assigned for waves of 9 m. The minimum and maximum values correspond to the upper limits of the Douglas Sea Scale Level 3 (slight waves) and Douglas Scale Level 7 (high waves) respectively. A description of the Douglas Sea Scale can be found in Owens (1982). The normalized values of this hazard indicator for each island are presented in Table A.1 for the historical reference period. For the projected future raw and normalized data are presented in Tables A.1.2-A.1.5.

**Extreme Wind (WiX98)**

Daily values of the model-derived wind speeds around each of the six selected islands (Cyprus, Crete, Malta, Corsica, the Canary Islands, and the Balearic Islands) were used for the calculation of the 98^th^ percentiles of this meteorological variable. The average of these values for each sub-period of analysis was used as the WiX98 hazard indicator. According to the regional climate simulations, for our historical reference period, extreme wind values range from 11.6 m/sec for the Canary Islands to 13.7 m/sec for Corsica (Table A.1). The normalization approach for this variable is similar to the one of WiX98, however, it is based on the Beaufort Wind Scale instead of the Douglas Sea Scale. In more detail, values of 0 were assigned for wind speeds of 9.3 m/sec, which is described as “fresh breeze” or Beaufort Scale 5, while values of 1 were assigned for wind speeds of 18.9 m/sec which is described as “gale” or Beaufort Scale 8. The projected future raw and normalized data are accordingly presented in Tables A.2-A.5.

**Mean Sea-Level Rise (MSLR)**

For the mean sea level rise, we considered by default zero values for the historical reference period for all islands. For the future, we employed the mean sea level rise projections based on ocean-atmosphere coupled simulations. Under RCP8.5, maximum MSLR (74 cm) is expected for the Canary Islands. The best-case scenario is for Cyprus where an MSLR value of about 20 cm is projected under pathway RCP2.6. The normalization of this indicator was based on expert judgement. We have assigned values of 0 for MSLR 0 cm and values of 1 for MSL of 100 cm, which is the top range of RCP8.5 global sea-level rise (IPCC 2013).

**Table A1** Values of risk indicators’ values for the Maritime Transport Impact Chain of “Risk of isolation due to transport disruption” for raw and normalized data, representative for the historical reference period 1986-2005. Fields of NA indicate that accurate information was not available.

**Table A2.** Same as Table A.1 for the mid-21^st^ century (2046-2065) under pathway RCP2.6.

**Table A3** Same as Table A.1 for the late-21^st^ century (2081-2100) under pathway RCP2.6.

**Table A4.** Same as Table A.1 for the mid-21^st^ century (2046-2065) under pathway RCP8.5.

**Table A5.** Same as Table A.1 for the late-21^st^ century (2081-2100) under pathway RCP8.5.

## A2. Exposure

**Islands’ Population (NPop)**

For the first exposure indicator involved in the risk of Maritime Transport disruption, we used the total population for each island (expressed in thousands of inhabitants). This indicator reflects the dependence of each island on maritime transport for importing goods and energy. It was derived from NUTS 2 and NUTS 3 EUROSTAT data. The normalization approach used was again the minimum/maximum methodology. Future population projections (years 2050 and 2090) were derived from the United Nations Department of Economic and Social Affairs (<https://population.un.org/wpp/>). This was the case for Cyprus and Malta given that they are both island countries. For the rest of the cases where island-based projections were not available, we assumed that the population trajectories will follow the trends of the countries to which they belong. Raw and normalized values are presented in Tables A.1-A.5.

**Number of passengers (NPax)**

For the second exposure indicator, we employed the annual total number of passengers per island (NPax). This includes permanent residents and tourists from and to the mainland, as well as, passengers in leisure cruises. NPax is derived from the European Union’s Statistical Office (EUROSTAT). Regional data are available for the NUTS 2 and 3 classifications. For our case studies, the EU island countries of Cyprus and Malta are included in NUTS 2, whereas the rest of the islands under investigation (Crete, Corsica, the Canary Islands, and the Balearic Islands) are included in the NUTS 3 classification. Since this indicator is dynamic through time, we have used the average number of the five more recent years available in the EUROSTAT database. The units of this indicator are thousands of passengers per year. According to the historical data, the greatest annual number of passengers (including cruises) is found for the Canary Islands, while the lowest number is found for Cyprus (Table A.1). To facilitate an inter-island comparison, we have used the minimum/maximum methodology, with a value of 0 assigned to the island with the lowest number of passengers and a value of 1 assigned to the island with the highest number of passengers. For the timescales discussed in the present study, no information about future projections was available for NPax. Nevertheless, we scaled the historical values for this indicator according to the population changes to provide a more realistic assessment for future risk.

**Value of Goods (VGTr)**

An important component of exposure is the total value of goods transferred by ships. Since it was difficult to obtain absolute numbers of values for islands, we have used as an alternative indicator the total freight (in tons). These were available per NUTS 2 and NUTS3 region covering all islands under investigation. A minimum/maximum normalization method was applied to facilitate the inter-island comparison. Values of this indicator were considered static for the future. According to the values of this indicator, the Canary Islands are the ones that are most dependent on Maritime Transport for the transfer of goods. Similarly, to the number of passengers, we have scaled the historical values of the value of goods according to the population change to provide a more realistic assessment for future risk.

**Number of Ports (NPor)**

The final exposure indicator that was used for the operationalization of the selected Impact Chain of the Maritime Transport sector is the number of ports per island or archipelago. Information on the number of ports was obtained through online resources^[[1]](#footnote-1),^^[[2]](#footnote-2),^^[[3]](#footnote-3),^^[[4]](#footnote-4),^^[[5]](#footnote-5),^^[[6]](#footnote-6)^. The normalization of this indicator was based on a rating scale that was defined by expert judgement (GIZ 2017). In more detail, higher sub-component risk (a value of 1) was assigned to islands that fell in the class [0,1], a value of 0.75 was assigned to islands that fell in the [2-3], a value of 0.50 was assigned to islands that fell in the class [4-5], a value of 0.25 was assigned to islands that fell in the class [6-7], and a value of 0 was assigned to islands or archipelagos that have more than 8 ports operating. This indicator was considered static for the future.

## A3. Sensitivity

**Renovated Infrastructure (NRni)**

This indicator is defined as the number of ports with critical infrastructures not renovated since 1993. For three of the considered islands (Cyprus, Malta and Corsica) there was available information that all ports have been partially and/or gradually renovated during the last decades. We assumed that this was the case for the three remaining case studies (Crete, the Canary Islands and the Balearic Islands). We, therefore, assigned values of 0 for this sub-component of sensitivity for all islands and all future scenarios. This sensitivity component did not affect our island intercomparison since equal values are assigned, however, we consider it in our analysis for potential use in follow-up studies.

**Increased costs of keeping ports' operability (ICost)**

Hazards related to climate change, mainly sea level rise (MSLR) will likely increase the cost of keeping ports operational. Such interventions will also have an impact on the transportation cost and also the prices of goods that are imported. Estimations of increased costs of keeping ports' operability under different scenarios of MSLR caused by climate change until 2100 are obtained from Leon et al., 2021). These costs have been estimated with reference to 1 meter; this is the investment needed to increase the infrastructures' height by 1 meter. There is not necessarily a strict correspondence between the MSLR and the required elevation of port infrastructures, which also depend on the coastal hydrodynamic and the shape of the dikes of each port. By experts' recommendation, we have assumed that a 1 m increase in port height is required to cope with the MSLR under the RCP8.5 scenario of emissions. Extrapolation for other RCP scenarios is then conducted based on proportionality.

## A4. Adaptive Capacity

**Percentage of Renewables (PER)**

This indicator of adaptive capacity describes the extent to which is each island is self-sustainable in terms of energy resources. For the present situation, the PER indicator values were extracted through online resources and regional reports^[[7]](#footnote-7),^^[[8]](#footnote-8),^^[[9]](#footnote-9)^. The island of Crete in Greece has the highest percentages of electricity provided by renewable energy sources (presently at 25%). On the other hand, the Balearic Islands has the lowest percentage of renewables in their energy production mix (currently 4.5%). These numbers are expected to increase for all islands due to the cost reduction and the increase in efficiency of the relevant technologies. Moreover, all EU countries are committed to adopting mitigation and decarbonization policies. In this context, for future pathways, we have used different scenarios. For the strong-mitigation pathway (RCP2.6), we assumed for all islands a "sustainability" pathway, for instance SSP1 from Riahi et al. 2017. This corresponds to values of PER at 50-60%. Noteworthy, this is a very conservative scenario since according to the EU’s long-term strategy (<https://ec.europa.eu/clima/policies/strategies/2050_en>), Europe will aim at being climate neutral by 2050. For the “business-as-usual” pathway (RCP8.5), we have considered a "regional rivalry" pathway similar to the SSP3 in Riahi et al. (2017). Under this pathway, the average contribution of renewable sources in total energy production is 25%. For the normalization of this indicator, we have used minimum/maximum values manually according to expert judgement. Based on information obtained from the Cyprus Organization for Storage and Management of Oil Stocks (<https://www.kodap.org.cy/>), we approximate that about 20% of the annual-total needs of crude oil and petroleum products are in stock at any time. We, therefore, assign an optimum risk value (0) when there is an 80% coverage of the needs of an island from renewables. In this context, a risk value of 1 is assigned for 0% renewable energy sources.

**Early Warning Systems (EWS)**

Information on the existence of Early Warning Systems was difficult to be obtained. Nevertheless, since all islands under investigation strongly depend on maritime means for the transportation of passengers and goods, we assumed that all ports are equipped with Early Warning Systems. Therefore, a value of 0 was assigned to this risk sub-component. This adaptive capacity component did not affect our island intercomparison since equal values are assigned, however, we consider it in our analysis for potential use in follow-up studies.

**Harbour Alternatives (NApt)**

This indicator is greatly relevant to the risk of Maritime Transport disruption. We considered as harbour alternatives the number of airports that can facilitate the transport of passengers or goods in the unfortunate case of a seaport closure. This number was obtained through online resources^[[10]](#footnote-10),^^[[11]](#footnote-11),^^[[12]](#footnote-12)^. The normalization of this indicator, which was considered as static for the future, was similar to the one applied for the number of ports. In more detail, it was based on a rating scale that was defined by expert judgement (GIZ, 2017). The highest sub-component risk (a value of 1) was assigned to islands that have 0 or 1 harbour alternatives, value of 0.75 were assigned to islands that fell in the [2-3] class, a value of 0.50 was assigned to islands that fell in the [4-5] class, a value of 0.25 was assigned to islands that fell in the [6-7] class, and a value of 0 was assigned to islands or archipelagos that have 8 or more airports operating. Future pathway RCP2.6 implies a decarbonization of the aviation sector to meet emission reduction targets. Nevertheless, this will probably remain predominantly powered by liquid fossil fuels (kerosene and bio- and synfuel derivatives) in the medium to long term because of limitations in electrical energy storage (Pagenkopf et al., 2019. Following the global scenarios of Gütschow et al. (2021), we considered a 10 and 40% reduction in the normalized indicator for harbour alternatives for the mid- and end-21st century periods.

**Potential for local agriculture and sustainable water resources (SPEI)**

The Standardized Precipitation-Evapotranspiration Index (SPEI) is a multi-scalar drought index based on climatic data (Begueria et al., 2014). It can be used for determining the onset, duration and magnitude of drought conditions with respect to normal conditions in a variety of natural and managed systems such as crops, ecosystems, rivers, water resources, etc. Here we use it as a proxy indicator for access to sustainable water resources and agriculture. Following Spinoni et al. (2018), we calculate SPEI at a 12-month accumulation scale, a good compromise between short timescales suitable for seasonal events and long timescales suitable for multi-annual cycles. Besides the historical simulation, we use the RCP2.6 and RCP8.5 future pathways. Monthly precipitation, maximum and minimum temperature data from the EURO-CORDEX and MENA-CORDEX initiatives were used. The calculation of potential evapotranspiration is based on the Thornthwaite method (Thornthwaite 1948). The SPEI calculation is based on the reference period of 1986-2005 to investigate if future conditions will deviate from this recent past period which is considered as the “normal” conditions. SPEI values between 0 and -1 were assigned normalized values of 0.25, values between -1 and -1.5 (moderately dry) as 0.5, values between -1.5 and -2 (very dry) as 0.75 and values lower than -2 (extremely dry) as 1.

**References**

Beguería S, Vicente-Serrano SM, Reig F, Latorre B (2014) Standardized precipitation evapotranspiration index (SPEI) revisited: Parameter fitting, evapotranspiration models, tools, datasets and drought monitoring. Int J Climatol 34:3001–3023. doi: 10.1002/joc.3887

GIZ (2017) The Vulnerability Sourcebook: Concept and guidelines for standardised vulnerability assessments. Deutsche Gesellschaft für Internationale Zusammenarbeit (GIZ)

Gütschow J, Jeffery ML, Günther A, Meinshausen M (2021) Country-resolved combined emission and socio-economic pathways based on the Representative Concentration Pathway (RCP) and Shared Socio-Economic Pathway (SSP) scenarios. Earth Syst. Sci. Data 13, 1005–1040. <https://doi.org/10.5194/essd-13-1005-2021>

IPCC (2013) Climate Change 2013: The physical science basis. Contribution of Working Group I to the Fifth Assessment Report of the Intergovernmental Panel on Climate Change [Stocker TF, Qin D, et al. (eds.)]. Cambridge University Press, Cambridge and New York.

Léon C et al. (2021) Downscaling climate change impacts, socio-economic implications and alternative adaptation pathways for islands and outermost regions. McGraw­Hill, Madrid, https://doi.org/10.5281/zenodo.5141549

OECD (2008) Handbook on Constructing Composite Indicators: Methodology and User Guide. Organisation for Economic Co-operation and Development (OECD)

Owens EH (1982) Sea conditions. In: Beaches and Coastal Geology. Encyclopedia of Earth Science. Springer, Boston, MA

﻿Pagenkopf, J., Van Den Adel, B., Deniz, Ö., Schmid, S., 2019. Transport transition concepts, in: Teske, S. (Ed.), Achieving the Paris Climate Agreement Goals: Global and Regional 100% Renewable Energy Scenarios with Non-Energy GHG Pathways for +1.5C and +2C. Springer International Publishing, Cham, pp. 131–159. https://doi.org/10.1007/978-3-030-05843-2_6

Riahi K, van Vuuren DP, Kriegler E, et al (2017) The Shared Socioeconomic Pathways and their energy, land use, and greenhouse gas emissions implications: An overview. Glob Environ Chang 42:153–168. doi: 10.1016/j.gloenvcha.2016.05.009

Spinoni J, Vogt J V., Naumann G, et al (2018) Will drought events become more frequent and severe in Europe? Int J Climatol 38:1718–1736. doi: 10.1002/joc.5291

Thornthwaite CW (1948) An Approach toward a Rational Classification of Climate. Geogr Rev 38:55. doi: 10.2307/210739

1. <https://www.cpa.gov.cy/en/home> [↑](#footnote-ref-1)
2. <https://www.transport.gov.mt/maritime-40> [↑](#footnote-ref-2)
3. <https://www.ynanp.gr/en/> [↑](#footnote-ref-3)
4. <https://www.bonifacio.co.uk/organisme/the-ports-in-corsica/> [↑](#footnote-ref-4)
5. <https://www.gobiernodecanarias.org/principal/> [↑](#footnote-ref-5)
6. <https://www.portsdebalears.com/en/> [↑](#footnote-ref-6)
7. <https://energy.gov.cy/> [↑](#footnote-ref-7)
8. <https://ec.europa.eu/eurostat> [↑](#footnote-ref-8)
9. <https://doi.org/10.1016/j.jclepro.2019.118356> [↑](#footnote-ref-9)
10. <https://airportcodes.aero/> [↑](#footnote-ref-10)
11. <https://www.icao.int> [↑](#footnote-ref-11)
12. <https://www.europe-airports.com/> [↑](#footnote-ref-12)
