# Supplementary material for: Maritime transport and regional climate change impacts in large EU islands and archipelagos
Source: EuroMediterr J Environ Integr. 2023 May 26:1–14. Online ahead of print. doi: 10.1007/s41207-023-00370-6 (PMC10213571; doi:10.1007/s41207-023-00370-6)
Supplement: Supplementary file 2 — Supplementary file2 (DOCX 508 KB) [file 41207_2023_370_MOESM2_ESM.docx]

# **APPENDIX B. Relative Risk Computation II: weighted aggregation of sub-components**

**Table B.1.** Operationalization of the Impact Chain of the Risk of Maritime Transport disruption for the control reference period.

**Table B.2.** Same as Table B.1 for the mid-21st century (2046-2065) and pathway RCP2.6.

**Table B.3.** Same as Table B.1 for the end of the 21^st^ century (2081-2100) and pathway RCP2.6.

**Table B.4.** Same as Table B.1 for the mid-21st century (2046-2065) and pathway RCP8.5.

**Table B.5.** Same as Table B.1 for the end of the 21^st^ century (2081-2100) and pathway RCP2.6.
